# Supplementary material for: Using traditional rhyme (folk song) as a tool for oral hygiene promotion (UTRATOHP) among children in rural communities in Nigeria: A protocol for a randomised controlled trial
Source: PLoS One. 2023 Jun 2;18(6):e0280856. doi: 10.1371/journal.pone.0280856 (PMC10237381; doi:10.1371/journal.pone.0280856)
Supplement: S2 File — (DOCX) [file pone.0280856.s002.docx]

**QUESTIONNAIRE**

| **Instructions** |
| --- |
| This questionnaire consists of **30** questions, divided into four sections (I -IV). The sections seek information on socio-demographics, oral hygiene practice, attitude, and knowledge. The questions only require you to tick the appropriate option corresponding to the question asked. The last section (Section V) will be completed by an examiner after examining your mouth. |
| ***N.B. Personal identification details are not required in order to ensure complete anonymity*** |

**Title**: **Using traditional rhyme (Folk Song) as a tool for oral hygiene promotion among children in rural communities in Nigeria: a protocol for a randomised controlled trial**

Serial Number ______

**SECTION I: BACKGROUND INFORMATION**

1. How old are you? (Age as at your last birthday in years) _______
2. What is your sex?
3. Male
4. Female
5. I prefer not to say
6. What type of school do you attend?
   1. Public
   2. Private
7. In a day, how much do you bring to school?
8. I usually do not bring money to school
9. Less than 100 naira
10. 100 – 200 naira
11. 201 – 500 naira
12. Above 500 naira
13. Are you living with your parents?
    1. Yes
    2. No
14. Are your parents living together?
    1. Yes
    2. No
15. What level of education did your father/ male guardian complete?
    1. No formal education
    2. Primary
    3. Secondary
    4. Post-secondary
    5. Tertiary
    6. I don’t know
16. What level of education did your mother/ female guardian complete?
    1. No formal education
    2. Primary
    3. Secondary
    4. Post-secondary
    5. Tertiary
    6. I don’t know
17. Father’s occupation?________________________________________________
18. Mother’s occupation? _______________________________________________

**SECTION II: ORAL HYGIENE PRACTICES**

|  | What do you use to brush your teeth? (Tick all the things you use from the options) | a. Toothbrush |  |
| --- | --- | --- | --- |
|  |  | b. Chewing stick |  |
|  |  | c. Cotton wool |  |
|  |  | d. Charcoal |  |
|  |  | e. My finger |  |
|  |  | d. Others: |  |
|  | How many times do you brush daily? | a. I don’t brush everyday |  |
|  |  | b. I usually brush once daily |  |
|  |  | c. I always brush at least twice daily |  |
|  | What texture of toothbrush do you use? | a. Soft |  |
|  |  | b. Medium |  |
|  |  | c. Hard |  |
|  | Does your toothpaste contain fluoride? | a. I don’t know what is fluoride |  |
|  |  | b. I don’t know if it has fluoride |  |
|  |  | c. Yes my toothpaste contains fluoride |  |
|  |  | d. No, my toothpaste does not contain fluoride |  |
|  | What is the name of toothpaste that you commonly use at home? |  |  |
|  | What is the quantity or size of toothpaste that you put on your toothbrush when you want to brush? | a. large size |  |
|  |  | b. small size |  |
|  |  | c. I can’t say if it is large or small |  |
|  |  | d. I don’t use toothpaste |  |
|  | What are the areas of your mouth that you brush during toothbrushing? (Tick all that apply to you) | a. My front teeth |  |
|  |  | b. My back teeth |  |
|  |  | b. My gums |  |
|  |  | c. My tongue |  |
|  | What technique (hand movement) do you use when you are brushing? | a. No particular motion/technique |  |
|  |  | b. I don’t know the technique/movement |  |
|  |  | c. horizontal motion/front and back movement |  |
|  |  | d. Up and down movement |  |
|  |  | e. Circular/round movement |  |
|  | Who brushes/cleans your mouth? (Tick all that apply to you) | a. I brush by myself |  |
|  |  | b. I brush under the supervision of an adult |  |
|  |  | c. An adult brushes for me |  |

**SECTION III: ATTITUDE TOWARDS ORAL HYGIENE PRACTICES**

| S/N |  | Strongly disagree | Disagree | Neither agree nor disagree | Agree | Strongly agree |
| --- | --- | --- | --- | --- | --- | --- |
|  | Cleaning your teeth is very important for the health of your mouth |  |  |  |  |  |
|  | Cleaning your teeth is very important for the health of your body |  |  |  |  |  |
|  | It is not important to brush your tongue |  |  |  |  |  |
|  | There is no problem if you do not brush twice daily |  |  |  |  |  |
|  | Even if your toothpaste does not contain fluoride, your teeth will still be very strong and will not have a hole |  |  |  |  |  |

**SECTION IV: KNOWLEDGE OF IDEAL ORAL HYGIENE PRACTICES**

|  | How many times should a child brush daily? | a. Once daily |  |
| --- | --- | --- | --- |
|  |  | b. At least twice daily |  |
|  |  | c. I don’t know |  |
|  | What texture of toothbrush is NOT good? | a. Soft |  |
|  |  | b. Hard |  |
|  |  | c. I don’t know |  |
|  | Is fluoride a constituent of good toothpaste? | a. Yes |  |
|  |  | b. No |  |
|  |  | c. I don’t know |  |
|  | What is the ideal quantity or size of toothpaste to be dispensed for tooth brushing at a time? | a. large size (fill the brush) |  |
|  |  | b. small size (pea-nut) |  |
|  |  | c. I don’t know |  |
|  | Is it important to brush your tongue when you are brushing your teeth? | a. Yes |  |
|  |  | b. No |  |
|  |  | c. I don’t know |  |
|  | Is Circular motion an ideal technique to use when brushing your teeth? | a. Yes |  |
|  |  | b. No |  |
|  |  | c. I don’t know |  |

**SECTION V: ORAL EXAMINATION**

**Teeth present:**

|  |  |
| --- | --- |
|  |  |

**ORAL HYGIENE STATUS**

**Simplified Oral Hygiene Index – Plaque/debris**

| 6 | 1 |  | 6 |
| --- | --- | --- | --- |
| 6 |  | 1 | 6 |

**Simplified Oral Hygiene Index – Calculus**

| 6 | 1 |  | 6 |
| --- | --- | --- | --- |
| 6 |  | 1 | 6 |

**Debri score________ Calculus score________ S-OHI score____________**

**Thank you for your time!**
